# Development of Solid Lipid Nanoparticles as Dry Powder: Characterization and Formulation Considerations

**DOI:** 10.3390/molecules28041545

**Published:** 2023-02-05

**Authors:** Debora Santonocito, Maria Grazia Sarpietro, Francesco Castelli, Maria Rosaria Lauro, Cristina Torrisi, Stefano Russo, Carmelo Puglia

**Affiliations:** 1 Department of Drug and Health Science, University of Catania, Viale Andrea Doria 6, 95125 Catania, Italy; 2 Department of Pharmacy, University of Salerno, Via Giovanni Paolo II 132, 84084 Fisciano, Italy

**Keywords:** solid lipid nanoparticles, spray-drying, freeze-drying, differential scanning calorimetry

## Abstract

Solid lipid nanoparticles (SLNs) are lipid-based colloidal systems used for the delivery of active compounds. Although SLNs have many benefits, they show important issues due to physical and chemical instability phenomena during storage. For these reasons, it is highly desirable to have a dried SLN formulation available. Therefore, the aim of the project was to identify suitable methods to obtain a dry powder formulation from an SLN suspension. The nanoparticle suspension was dried using both freeze- and spray-drying techniques. The suitability of these methods in obtaining SLN dry powders was evaluated from the analyses of nanotechnological parameters, system morphology and thermal behavior using differential scanning calorimetry. Results pointed out that both drying techniques, although at different yields, were able to produce an SLN dry powder suitable for pharmaceutical applications. Noteworthily, the freeze-drying of SLNs under optimized conditions led to a dry powder endowed with good reconstitution properties and technological parameters similar to the starting conditions. Moreover, freeze–thaw cycles were carried out as a pretest to study the protective effect of different cryoprotectants (e.g., glucose and mannitol with a concentration ranging from 1% to 10% *w*/*v*). Glucose proved to be the most effective in preventing particle growth during freezing, thawing, and freeze-drying processes; in particular, the optimum concentration of glucose was 1% *w*/*v*.

## 1. Introduction

We are currently living in the golden age of pharmaceutical nanocarriers, and we are witnessing a maturation stage of the original idea of nanotechnology applied to the formulation of innovative pharmaceutical carriers. We are aware that nanoformulations are extremely valuable tools for drug delivery applications, and the vaccination against COVID-19 has been an important test case. The current challenge is how to optimize the nanocarriers to ensure that they are safe, effective, and scalable, so that they can be manufactured at an industrial level and advance to clinical use. 

In this context, lipid nanoparticles have gained ground; the usage of these systems has brought important innovations in the pharmaceutical field [1,2,3], as they show many advantages such as low cost, high-scale production, chemical versatility, and bioacceptability due to the use of GRAS (Generally Recognized as Safe) ingredients [4,5,6,7]. In particular, they are able to achieve a targeted and prolonged release, avoiding rapid drug degradation over time [8,9,10]. 

One of the key challenges associated with the development of SLN preparation is to remove the high amount of water (70–95%) in order to achieve maximum physical and chemical stability [11,12,13,14] and to optimize the formulation to be administered through specific routes such as the pulmonary route of administration [15]. 

Spray- and freeze-drying are the most used techniques to convert liquid nanosuspension into dry nanoparticles.

Spray-drying, the less expensive technique, is widely used to convert a liquid into a dry system in a one-step process and to achieve fine powders [16]. This technique is widely used in pharmaceutical field since it is rapid, low-cost, continuous, and scalable for the production of dry powders [17,18]. 

While the physical state of a solid in suspension is not affected by high temperatures during the spray-dying process, drying of solid lipid nanoparticles can be more challenging because they may melt during the process [19].

In some studies reported in the literature, the spray-drying of SLN from different lipid types, such as tristearin and Compritol 888 ATO, was evaluated [20,21,22]. Interestingly the results showed that the particles could be embedded in matrices without losing their nanoparticulate properties. The authors of these studies assumed that the temperatures to which the particles were exposed were well below their melting temperatures and, thus, did not have an effect on the physical state of the lipids during drying.

Another strategy to overcome the problem of excessive heating is the use of an organic solvent in order to decrease the temperature required for solvent evaporation. However, these solvents are highly flammable; therefore, their use is severely limited.

On the other hand, freeze-drying is commonly used to convert lipid emulsions into solids of sufficient physical stability for distribution and storage. The process involves the removal of water from the SLN by the aid of sublimation and desorption under vacuum. Freeze-drying offers many advantages, such as long-term physical and chemical stability, and ease of reconstitution, while the biggest shortcoming of the method is the increase in particle size due to aggregation of lipid nanoparticles [23].

In order to overcome this important limitation, cryoprotectants, compounds able to immobilize the nanocarriers within their glassy matrix, have been successfully used in preventing the aggregation of the nanoparticles and protecting them against the mechanical stress of ice crystals. The most used cryoprotectants in freeze-drying of lipid-based nanoparticles are sugars such as glucose and mannitol [24,25,26]. The stabilization effect of these substances depends on their concentration, and it has been observed that an increase in the cryoprotectant concentration beyond a certain level could destabilize the nanoparticle suspension [27,28]. 

On the basis of previous considerations, the aim of the work was the evaluation of freeze- and spray-drying techniques as methods to obtain a dry powder formulation from a model SLN suspension.

Both methods required the optimization of the experimental parameters in order to obtain powders with acceptable physicochemical and structural characteristics. 

With regard to the lyophilization process, the most used cryoprotectants at different concentrations were evaluated to obtain stable dried nanoparticles with a short reconstitution time in water. 

Some important parameters involved in the spray-drying technique were optimized to obtain a SLN dry powder characterized by suitable physicochemical characteristics. In particular, the best condition to avoid both aggregation phenomena on the drying chamber walls and the fusion of lipid nanoparticles due to high temperatures was evaluated. 

The suitability of these two different techniques to produce a dry SLN powder was evaluated through an analysis of the nanotechnological parameters (mean particle size, polydispersity index, and zeta potential), determined before and after the drying processes, and through an analysis of the system morphology by fluorescence microscopy (FM) and transmission electron microscopy (TEM). In particular, the first technique was shown to be the most suitable to characterize the spray-dried batches, while the second was applied to evaluate the morphology of the lyophilized samples. Lastly, the evaluation of the thermal behavior of the nanoparticle suspension, determined before and after drying, gave us important indications regarding the process effectiveness.

## 2. Results and Discussion

### 2.1. Influence of Spray-Drying Parameters on Yield Value

Multiple studies in the scientific literature have provided many examples regarding the importance of spray-drying in nanotechnological processes [29,30]. This technique has demonstrated its ability to optimize the particle size, shape, surface roughness, and surface composition of polymeric nanoparticles by modifying critical process parameters and formulation variables.

Unfortunately, the application of this drying technique to lipid nanoparticle suspensions is not a simple task. In fact, it can potentially cause aggregation or fusion of the nanoparticles due to high temperatures and shear forces. Furthermore, high temperatures may also be responsible for drug degradation phenomena, thus causing a reduction in/loss of drug therapeutic activity. A potential strategy to control these unfavorable features is represented by the modification, during the drying process, of some important parameters such as the feed composition, the inlet (T_in_) and outlet (T_out_) temperatures, and the aspiration rate (ASP) [31]. A scientific study reported in literature demonstrated the key role of T_in_ in influencing the physical state of the lipids during drying [32]. The authors of this study pointed out that the temperatures to which the particles were exposed were well below the melting temperatures of the solid lipids, thereby obtaining a dry powder with suitable features. In another study, Freitas and Müller demonstrated that a melting temperature of at least 65 °C is necessary to obtain a valid SLN dry powder from an aqueous suspension [22]. The authors hypothesized that, during spraying, the evaporated moisture produced a film around the droplets, able to absorb most of the heat. This could explain why no increase in temperature or consequent degradation of the heat-sensitive materials was observed. The use of an ethanol–water mixture as feed solution is a strategy used to decrease the temperature required for solvent evaporation [22].

In the present work, we formulated aqueous SLNs dispersions (batch D) and hydroalcoholic SLNs dispersions (batches A, B, and C) using the lipid Compritol 888 ATO, which has a melting point of about 72 °C, in order to keep the process temperatures as low as possible. The results expressed as process yield (RP%) are reported in Table 1. Batches A_1_ and A_2_ showed the best results in terms of RP%: 45.0 ± 0.5 and 58.33 ± 0.9, respectively. The differences observed between the two batches refer to the aspiration rate values. As a general rule, a higher ASP% value was responsible for the aspiration of the smaller lipid nanoparticles, while a low aspiration rate prolonged the residence time of the nanoparticles in the apparatus chamber. Strategically the ASP% values were set around 70% and 50%, thus avoiding the total aspiration of the nanoparticles.

Furthermore, the results clearly pointed out that the difference between T_in_ and T_out_ affected the residual moisture of the product; hence, a higher difference between the two values correlated to more residual moisture. All hydroalcoholic SLN suspensions were processed at 60 °C, with a T_out_ of around 30–35 °C and a maximum temperature load of 15–20 °C. Instead, the aqueous SLN suspensions were processed at 110 °C, reaching an outlet of 69–70 °C, with a maximum internal temperature of 49–50 °C. The addition of ethanol enabled the lowering of T_in_ values (from 110 °C to 60 °C); upon increasing the water percentage in the feed, we observed a noteworthy decrease in RP%, probably due to the residual moisture. These results are in accordance with data reported in the literature [22].

#### Characterization of Spray-Dried Nanoparticles

The characterization of nanoparticles was performed using photon correlation spectroscopy (PCS). As reported in Table 2, the technological parameters of the spray-dried nanoparticles changed considerably compared to the initial conditions. A slight increase in the polydispersity index (PDI) was observed, while the particle size showed values of ~1 μm due to the formation of macroaggregates during spraying. With regard to the zeta potential (ZP), the results showed positive values due to the positive charge of the added surfactant. 

**Figure 1:**
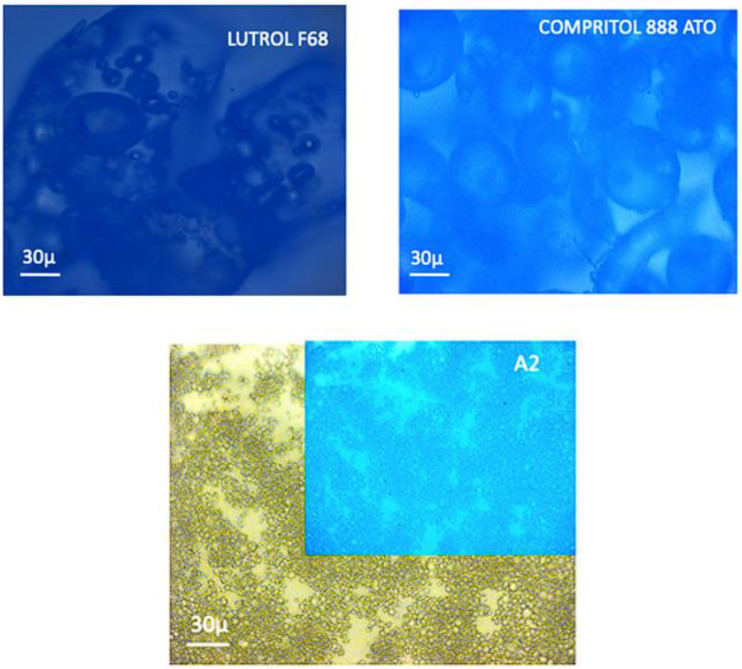
Fluorescence microscopy (FM) micrographs of raw materials, Lutrol F68 and Compritol 888 ATO (4′,6-diamidino-2-phenylindole; DAPI, 40×) and A_2_ batch (DAPI, blue image, and brightfield, 40×).

Figure 1 shows the results of the fluorescence microscopy analysis carried out on the raw materials (Lutrol F68 and Compritol 888 ATO) and on batch A_2_, which gave the best result in terms of RP% value.

Lutrol F68 appeared as a mixture of crystals and amorphous spherical particles with sizes around 30 μm, while Compritol 888 ATO consisted of spherical particles of about 100 μm (Figure 1). Noteworthily, batch A_2_ was characterized by well-structured spherical nanoparticles with a mean diameter of around 1 μm, while no traces of initial raw materials were present. These results are in line with the PCS analyses. 

**Figure 2:**
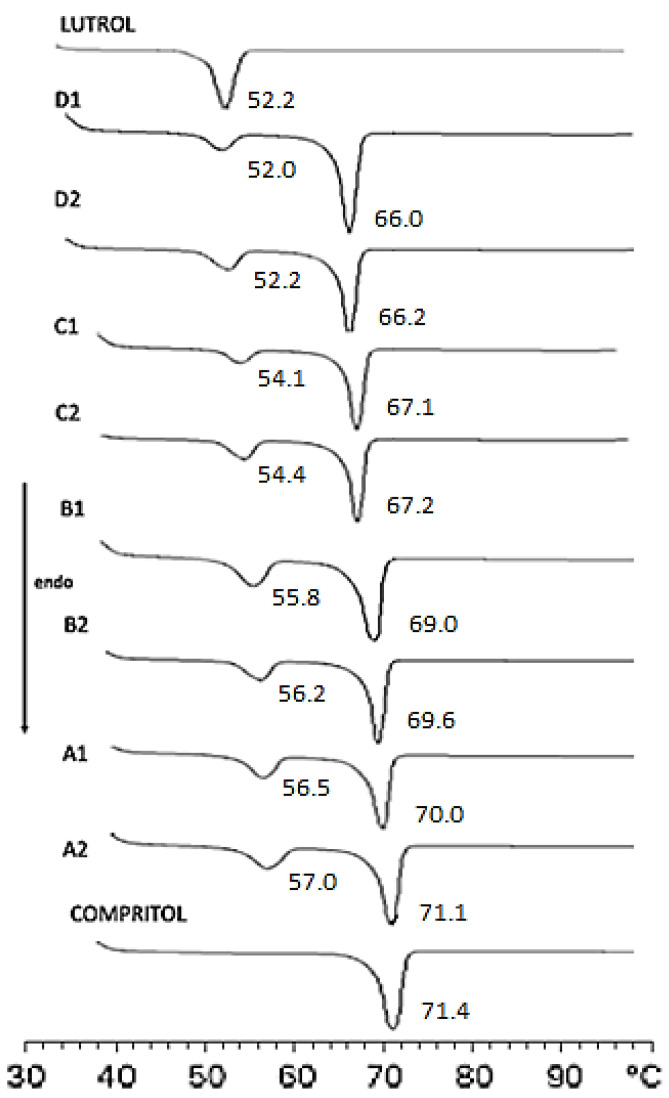
Differential scanning calorimetry of raw materials (Lutrol F68; Compritol 888 ATO) and processed batches (A_1_, A_2_, B_1_, B_2_, C_1_, C_2_, D_1_, and D_2_) of SLNs. The number next to each peak represents the peak temperature (expressed as °C).

DSC thermograms of spray-dried SLNs were carried out following the program described in Table 8 of Section 3. Results showed that batches A_1_ and A_2_ (60%:40% *w*/*w* EtOH:H_2_O) were well-structured compared to others. In fact, the peak intensity relating to the Compritol 888 ATO was decreased, probably due to the different forms of crystallization of the nanostructured lipid (Figure 2) [33].

### 2.2. Characterization of Lyophilized Nanoparticles 

**Figure 3:**
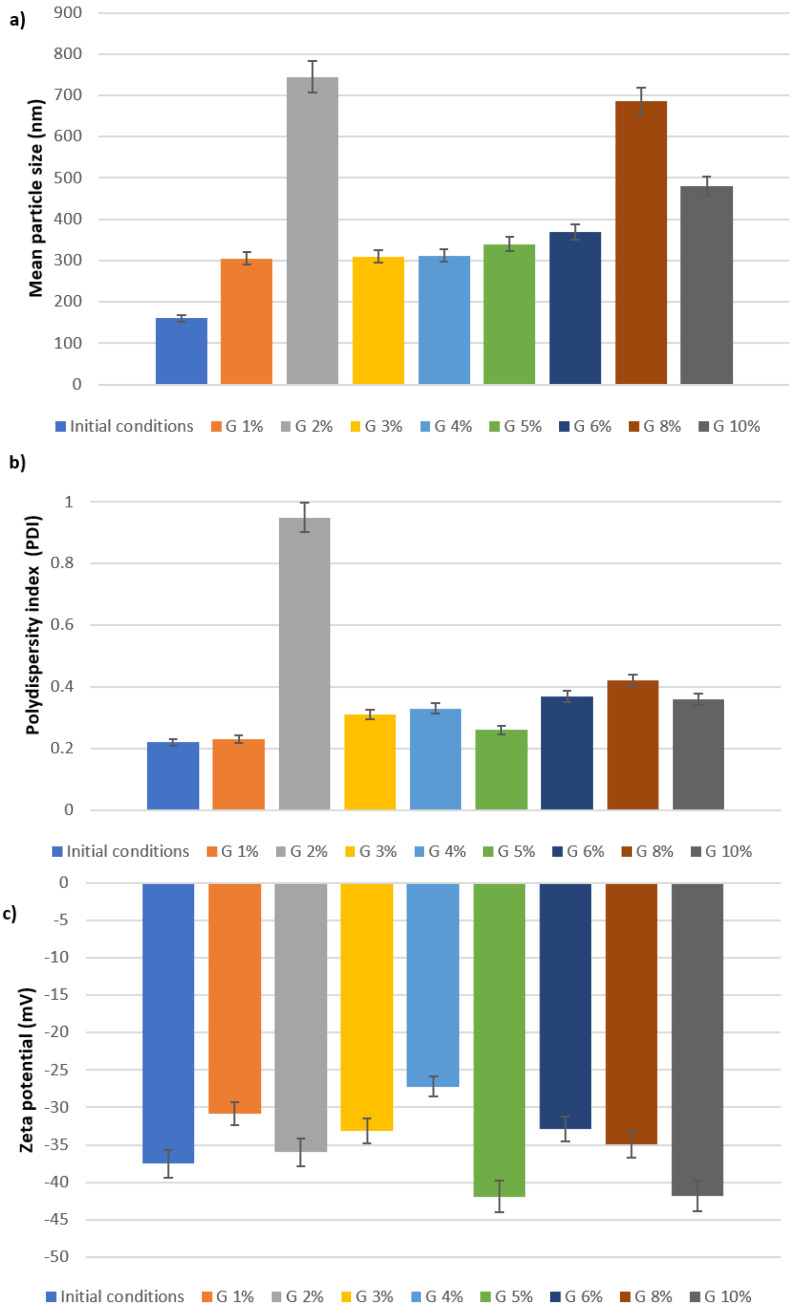
(**a**) Mean particle size (Z-Ave), (**b**) polydispersity index (PDI), and (**c**) zeta potential of SLNs using increasing concentrations of glucose.

The aqueous SLN dispersions were processed for the lyophilization. The maintenance of a nanotechnological parameters, particularly the nanoparticle size, after freeze-drying is considered a good indication of a successful freeze-drying cycle [34,35]. Therefore, we paid attention to select the optimal reconstitution method and cryoprotectant concentration, to obtain stable dried nanoparticles with a short reconstitution time in water. Many methods could be used to achieve the resuspension of freeze-dried nanoparticles as manual shaking, vortexing, or sonication. Usually, freeze-dried products are rehydrated immediately after the addition of water, although a long reconstitution time could be obtained as in the case of collapsed formulations [36]. In this preliminary study, manual shaking was chosen as the simplest reconstitution method. The results demonstrated that mannitol in different concentrations (1–10% *w*/*v*) were not effective as cryoprotectant. In fact, all samples dispersed after lyophilization showed a significant increase in both mean particle size, ranging from 308 to 1293.9 nm, and PDI values that were around 1.000. On the contrary, very promising results were obtained using glucose as cryoprotectant at the same tested concentrations. These data are in agreement with the literature [14]. As reported below (Figure 3), the data demonstrated that concentrations between 1% and 4% (*w*/*v*) glucose (with the exception of 2%) were the best to obtain a cryoprotectant effect for SLNs during lyophilization.

The results showed that freeze-drying of SLNs, under optimized conditions, produced a dry powder with good reconstitution properties; moreover, the technological parameters of post-freeze-dried SLNs were similar than of the initial conditions. 

**Figure 4:**
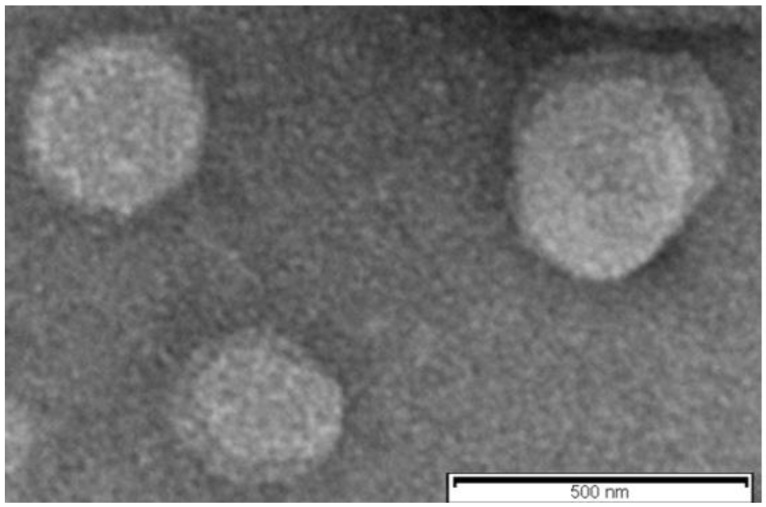
Transmission electron microscopy (TEM) of reconstituted SLN (sample G_1_).

The morphology of reconstituted SLN (sample G_1_) was investigated by TEM (transmission electron microscopy) to corroborate the previous data (Figure 4). In agreement with the PCS analyses, TEM image showed that the lipid nanoparticles had a spherical appearance with a particle size around 350 nm.

#### Differential Scanning Calorimetry (DSC)

Differential scanning calorimetry (DSC) was employed to assess the thermotropic behavior of raw materials (Compritol 888 ATO and Lutrol F68) and SLNs before, during, and after lyophilization.

**Figure 5:**
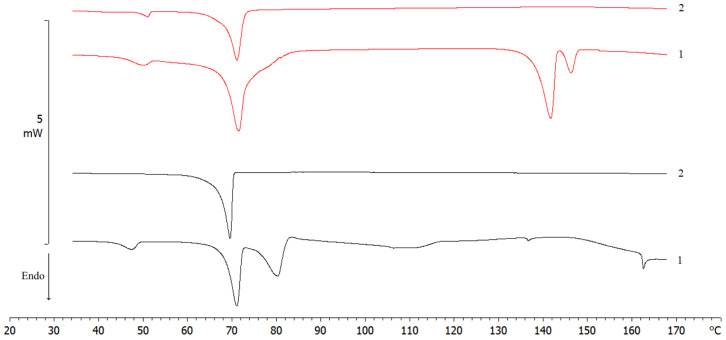
DSC thermograms of G_1_ lyophilized SLN using the technique of inverted lid (black) or pierced lid (red) technique.

According to PCS data, glucose appears to be an effective cryoprotectant for SLN. In order to prevent the caramelization of glucose [37] and its incorporation into SLNs by interfering with thermal peaks relating to SLNs, attention was paid to select the most suitable scanning program. SLNs freeze-dried with the addition of 1% *w*/*v* glucose (SLN G_1_) were chosen as the reference concentration for this preliminary study. The scanning program (Table 7 of Section 3), consisting of a heating scan from 25 °C to 170 °C and a cooling scan from 170 °C to 25 °C (Figure 5), was chosen to observe the formation and the behavior of the caramel due to the high process temperature. Moreover, the scanning program was performed using two different experimental conditions: pierced or inverted lid [38,39]. The thermotropic parameters are reported in Table 3.

In the inverted lid technique, the first scan showed broad and poorly resolved peaks, probably due to the formation of a caramel-like product derived from the reaction between glucose and residual humidity [37]. Moreover, the main peak relating to glucose moved to lower temperature (80 °C), while other small peaks were observed during all temperature scans up to 170 °C. From the second scan thermogram, the calorimetric peak of Compritol 888 ATO appeared broader than its original morphology due to the transformation of bulk lipid into SLNs, while all other signals disappeared.

In the pierced lid procedure, the first scan showed a double peak relating to glucose due to its polymorphic variation induced by freeze-drying and interaction with SLNs [40,41]. Moreover, it was observed that glucose exerted less of an effect on Lutrol F68 and Compritol 888 ATO peaks. In the second scan, these last peaks were preserved, while the calorimetric peak of glucose disappeared due to its melting and decomposition. Subsequently, this technique was adopted to evaluate all freeze-dried SLN samples using another scanning program (Table 8, Section 3). The scanning program, consisting of a heating scan from 25 °C to 85 °C and a cooling scan from 85 °C to 25 °C, was chosen to observe the thermal behaviors between SLN and raw materials (Lutrol F68 and Compritol 888 ATO). 

**Figure 6:**
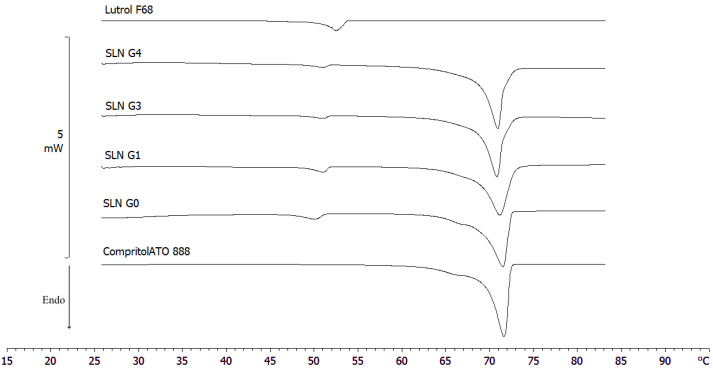
DSC thermograms of raw Compritol 888 ATO, Lutrol F68 and lyophilized SLN samples.

Furthermore, calorimetric curves of freeze-dried SLNs without and with glucose at different concentrations (1%, 3%, and 4% *w*/*v*) were performed to demonstrate the importance of cryoprotectant during lyophilization process and select the most suitable concentration (Figure 6, Table 4).

The calorimetric curve of raw Compritol 888 ATO showed a sharp peak at 72 °C, corresponding to its melting point, and a shoulder at about 65 °C; instead, the peak relating to the raw Lutrol F68 was observed at 52 °C.

The curve of freeze-dried SLNs with cryoprotectant at 1% *w*/*v* (SLN G_1_) showed that Lutrol F68 signal was similar to raw Lutrol F68 in term of morphology and peak temperature with an enthalpy comparable to G_0_. On the contrary, the Compritol 888 ATO signal was similar to G_0_ with the exception of the pre-transition shoulder, which appeared thinner.

The curves of freeze-dried SLNs with cryoprotectant at 3% and 4% *w*/*v* (SLN G_3_ and SLN G_4_) showed that the Compritol 888 ATO signal had a sharp peak at 72 °C and a post-transition shoulder at 73 °C; instead, the Lutrol F68 signal completely disappeared.

**Figure 7:**
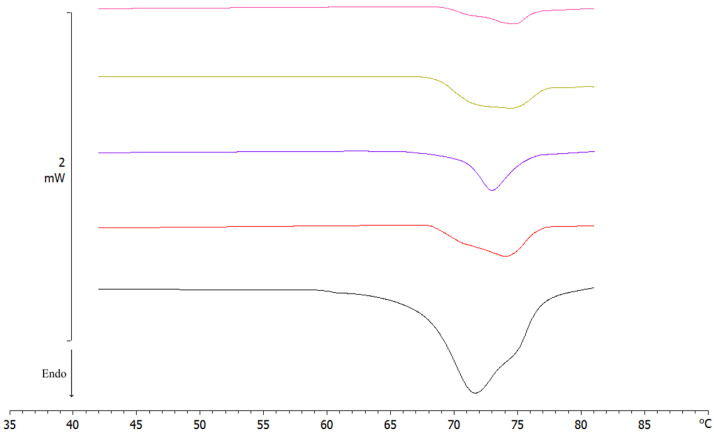
DSC thermograms of pre- and post-lyophilized SLNs. Pre-lyophilization SLN (black); SLN G_0_ (red); SLN G_1_ (blue); SLN G_3_ (yellow); SLN G_4_ (pink).

As reported in the literature, these results demonstrated that glucose interacted with SLNs, reducing both the enthalpy and the peak temperatures of the signal attributable to Compritol in a dose-dependent manner. This action was due to several defensive mechanisms of the cryoprotectant, such as the reduction in the cooperation of lipid components and the formation of a high-viscosity glass matrix that prevents damage by ice crystals [42]. However, it was interesting to note that the morphology of G_3_ and G_4_ peaks was different from that of the initial peak; this could have been due to possible damage caused by higher concentrations of cryoprotectant [43]. Moreover, PCS data proved that 1% glucose was the most effective concentration in preventing particle growth during freezing. We confirmed this result by reconstitution studies. Briefly, we diluted the freeze-dried products with the same volume of water lost during the lyophilization process, and then we shook the mixture for a minute. In order to underline the key effect of the cryoprotectant on the crystalline structure of the nanoparticles during freeze-drying, the calorimetric curves of pre- and post-lyophilized SLNs were compared (Figure 7, Table 5).

All post-freeze-dried samples, except SLN G_1_, showed low enthalpy and calorimeter curves completely changed compared to pre-freeze-dried SLNs. This could have been due to polymorphism and the addition of gradually higher glucose concentrations [40,41].

As previously reported, there are many methods to achieve the resuspension of freeze-dried nanoparticles, such as manual shaking, vortexing, or sonication. Therefore, we compared and evaluated them in order to choose the most suitable method to obtain stable nanoparticles with a short reconstitution time in water.

**Figure 8:**
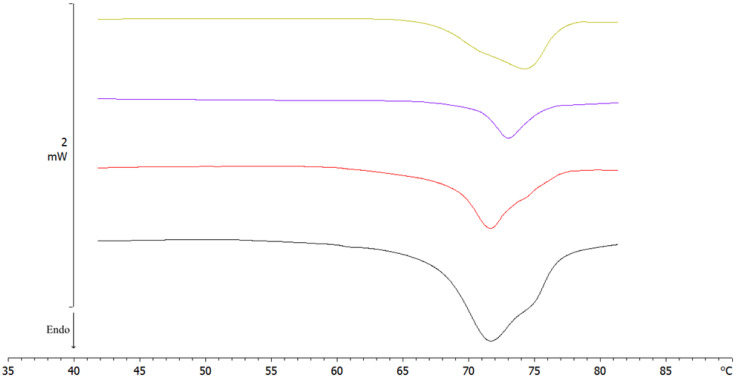
DSC thermograms of SLN G1, resuspended with three different methods, compared to pre-lyophilization SLN. Pre-lyophilization SLN (black); ultrasonicated SLN G1 (red); manually shaken SLN G1 (blue); vortexed SLN G1 (yellow).

The influence of the resuspension method on the thermotropic behavior of SLN G_1_ was performed using the previous program (Table 9 of Section 3) comparing manual shaking, 24 h long vortexing, and ultrasonication (2 min, cycle 1, 100% amplitude) (Figure 8, Table 6).

SLN G_1_ ultrasonicated showed a main peak at 71.64 °C and a shoulder at a higher temperature, while the thermotropic morphology of SLN G_1_ vortexed was completely different, showing a broad shoulder around 70 °C and a main peak at 74 °C. Instead, SLN G_1_ manually shaken possessed the main peak at a higher temperature than pre-lyophilization SLN, as well as a lower enthalpy than SLN resuspended using other methods. 

Although vortex and ultrasonication methods showed a better enthalpy than manual shaking, ultrasonication proved to be the best method as the thermogram was perfectly superimposable on that of the pre-freeze-dried sample in terms of peak temperature (71.68 vs. 71.64 °C) and morphology (a slight pre-transition derivative and a post-transition concave shoulder). This could have been due to the low HLB index (about 3) and high melting point of Compritol 888 ATO lipid, which require strong mechanical and thermal conditions (ultrasonication) to achieve good resuspension [44,45].

## 3. Materials and Methods

### 3.1. Materials

3,3′-Diaminobenzidine (DAB), mannitol, glucose, and ethanol were purchased from Merck (ST. Louis, MO, USA). Pluronic F68 (poloxamer 188) was purchased from BASF (Florham Park, NJ, USA), and Compritol 888 ATO, a mixture of mono-, di-, and triglycerides of behenic acid, was obtained from Gattefossè (Milan, Italy). Lutrol F68 was provided by BASF ChemTrade GmbH (Burgbernheim, Germany).

### 3.2. Preparation of SLNs

SLNs were prepared using the ultrasonication method [46,47]. Briefly, 0.8% *w*/*v* Compritol 888 ATO was melted at 80 °C (10 °C more than lipid melting point) and then was dispersed in hot (80 °C) surfactant solution (Lutrol F68; 0.1% *w*/*v*) using a high-speed stirrer (UltraTurrax T25; IKA-Werke GmbH & Company KG, Staufen, Germany) at 24,000 rpm for 8 min. The obtained pre-emulsion was ultrasonicated for 4 min using a UP 400 S Ultraschallprozessor (Dr. Hielscher GmbH, Teltow, Germany), maintaining the temperature at least 4 °C above the lipid melting point. The hot dispersion was then cooled in an ice bath under high-speed homogenization (UltraTurrax T25; IKA-Werke GmbH & Company KG) at 4000 rpm for 5 min. The post-sonication dilution was carried out by gradually increasing the water amount with a water/ethanol mixture at different *v*/*v* ratio (60:40, batch A; 50:50, batch B; 40:60, batch C) or with 100% *v*/*v* water (batch D). 

### 3.3. Characterization and Morphology of SLNs 

The nanoparticle size and the polydispersity index (PDI) were measured by photon correlation spectroscopy (PCS) using a Zeta Sizer Nano-ZS90 (Malvern Instrument Ltd., Worcs, UK) [48]. Analyses were performed using a 90° scattering angle at 20 ± 0.2 °C. The zeta potential (ZP), an indicator of the stability of a dispersed system, was measured by electrophoretic light scattering (ELS) using the same instrument. All samples were prepared by diluting 100 μL of SLN suspension with 900 μL of deionized water. Each value represents the average of three determinations. 

Two different methods were used to study the morphology of dried suspension: fluorescence microscopy (FM) and transmission electron microscopy (TEM). The first was used to characterize the spray-dried batch (sample A_2_) and the second was used to evaluate the morphology of sample G_1_, obtained from lyophilization.

Lutrol F68, Compritol 888 ATO, and sample A_2_ were observed with a Zeiss Axiophot fluorescence microscopy (FM) apparatus, with 40 × 1.4 NA plan Apochromat oil immersion objectives (Carl Zeiss Vision, München-Hallbergmoos, Germany) using brightfield or standard DAPI (4′,6-diamidino-2-phenylindole) optics that adsorb violet radiation (max 372 nm) and emit a blue fluorescence (max 456 nm).

Lyophilized sample G_1_ was evaluated by transmission electron microscopy (TEM) using a Philips EM 400T microscope (Eindhoven, The Netherlands). Briefly, a drop of reconstituted sample was deposited on the surface of a 200 mesh Formvar^®^-coated copper grid (TAAB Laboratories Equipment, Ltd., Aldermaston, UK). After evaporation, the specimen was sprayed with chromium prior to imaging (Quorum Q150T ES East Grinstead, West Sussex, UK). Coating was carried out at 120 mA for 30 s.

### 3.4. Spray-Drying of Nanoparticles 

The spray-drying process was performed in a spray-dryer laboratory-scale (Buchi Mini Spray-Dryer B-191, Buchi Laboratoriums-Tecnik, Flawil, Switzerland). Three different spray-drying variables were applied to optimize the dried process as follow: feed composition (EtOH:H_2_O 60:40, EtOH:H_2_O 50:50, EtOH:H_2_O 40:60, or 100% H_2_O); inlet temperature (T_in_; 60 °C or 110 °C for alcoholic or aqueous feed, respectively); aspirator rate (Asp; 50, 70, or 100). The standard spray-drying parameters were as follows: nozzle diameter (d; 0.7 mm); air pressure (p; 6 atm); spray flow rate (Φ, 3 mL/min); drying air flow rate (500 L/h). The outlet temperature (T_out_) changed from 25 to 100 °C as a function of T_in_. Each preparation was carried out in triplicate. All spray-dried nanoparticles were collected and stored under vacuum for 48 h at room temperature. The reconstitution ability of this technique was evaluated by redispersing 20 mg of each batch in 25 mL of deionized water; then, 0.01% of surfactant and DAB (0.01%) were added. The mixture was stirred for 5 min, vortexed for 5 min, and finally, ultrasonicated for 2.5 h in an ice bath.

### 3.5. Freeze-Drying of Nanoparticles 

Lyophilization of SLNs was preceded by a pre-formulation study necessary to establish the cryoprotectant most suitable for the formulation. Through accurate bibliographic studies, it was possible to notice that the ideal cryoprotectants are glucose and mannitol [24,25,26]. After choosing the ideal excipients to obtain a good freeze-dried product, the attention was paid to the optimization of their concentrations. 

SLN dispersions were added with the cryoprotectant (1–10% *w*/*v*) before freezing. After freezing, 2 mL of nanosuspension was lyophilized for 24 h. In order to rehydrate the lyophilized nanoparticles, the same volume of water lost during lyophilization was added. After reconstitution by manual shaking, nanotechnological parameters of SLNs were measured by PCS. 

### 3.6. Differential Scanning Calorimetry (DSC)

The experiments were carried out using a DSC 822^e^ calorimeter by Mettler Toledo (Greifensee, Switzerland). A Mettler TA STAR^e^ software (version 16.00) was used to analyze the data. The calorimeter was calibrated using Indium (99.95%), according to the settings of the instrument. The sensitivity was automatically chosen as the maximum possible by the calorimetric system. Aluminum crucibles of 40 µL for dried SLNs and 160 µL for nanoparticle suspensions were used [49,50]. 

Samples were submitted to different scanning programs as reported in Table 7, Table 8 and Table 9.

### 3.7. Statistical Analysis

Statistical data analysis was performed using Student’s *t*-test.

## 4. Conclusions

Spray-drying and lyophilization are suitable methods to produce a dry powder from a lipid nanosuspension, although with a different yield and with a complexity due to the presence of different variables related to the instrument (T_in_, T_out_, and ASP) and/or the formulation (kind of cryoprotectant and concentration of employ). 

The aim of the present work was not to compare the two methods or to define the best one, but to set for both the best conditions to produce a valid dried powder from an SLN suspension. The results showed that both methods, suitably improved, could be used for this aim. The evaluation of the nanotechnological parameters, and the results from the morphological studies corroborated these findings. 

Further evidence arising from the present study refers to the lyophilization technique that, under optimized conditions, can guarantee an SLN dry powder with good reconstitution properties. In particular, the cryoprotectant glucose, at the concentration of 1% *w*/*v*, proved to be most effective in preventing particle growth during the freeze-drying process. 

## Figures and Tables

**Table 1:** Variables and yield of the spray-drying process. d (µm): nozzle diameter; T_in_: inlet temperature; T_out_: outlet temperature; p: air pressure; ASP: aspiration; *Ψ*: flow rate; Rp: process yield; V: sprayed volume; SD: standard deviation.

*Batch*	*Feed* (% *w/w*) *EtOH:H_2_O*	*V* (mL)	*d* (µm)	*P* (atm)	*T_in_* (°C)	*T_out_* (°C)	*ASP* (%)	*Ψ* (mL/min)	*Rp* (%) ± *SD*
**A_1_**	60:40	100	700	6	60	29–30	70	3	45.0 ± 0.5
**A_2_**	60:40	100	700	6	60	25–26	50	3	58.33 ± 0.9
**B_1_**	50:50	100	700	6	60	32–33	70	3	43.71 ± 0.2
**B_2_**	50:50	100	700	6	60	34–35	50	3	56.82 ± 0.2
**C_1_**	40:60	100	700	6	60	34–35	70	3	38.16 ± 0.2
**C_2_**	40:60	100	700	6	60	34–35	50	3	44.31 ± 0.1
**D_1_**	0:100	100	700	6	110	69–70	70	3	21.0 ± 0.1
**D_2_**	0:100	100	700	6	110	69–70	50	3	24.84 ± 0.2

**Table 2:** Technological parameters of the pre-spray-dried and post-spray-dried samples.

Batches	Average Size (nm)	PDI	ZP (mV)
**Initial conditions**			
**A_1_**	228.2	0.278	−28.2
**A_2_**	220.0	0.250	−31.5
**B_1_**	234.5	0.264	−28.4
**B_2_**	237.1	0.283	−28.3
**C_1_**	209.5	0.252	−32.1
**C_2_**	221.9	0.300	−36.71
**D_1_**	159.1	0.268	−37.8
**D_2_**	167	0.253	−38.2
**After spray-drying**			
**A_1_**	1000	0.362	+48.0
**A_2_**	1000	0.266	+22.2
**B_1_**	1000	0.368	+46.3
**B_2_**	1000	0.282	+39.3
**C_1_**	1000	0.280	+62.8
**C_2_**	1000	0.351	+42.2
**D_1_**	1000	0.343	+43.0
**D_2_**	1000	0.301	+50.8

**Table 3:** Enthalpy variations (ΔH) and peak temperatures of the first scan and second scan of G_1_ lyophilized SLN obtained through inverted lid and pierced lid procedures.

Sample	ΔH (J/g) Compritol 888 ATO	ΔH (J/g) Lutrol F68	ΔH (J/g) Glucose	Peak T (°C) Compritol 888 ATO	Peak T (°C) Lutrol F68	Peak T (°C) Glucose
**Pierced lid scan 2**	−113.63	−49.71	----	71.16	50.99	----
**Pierced lid scan 1**	−108.98	−108.98	−119.71; −22.88	71.51	50.05	141.73; 146.31
**Inverted lid scan 2**	−120.22	----	----	69.60	----	----
**Inverted lid scan 1**	−98.52	−140.29	−76.29	71.07	47.42	80.32

**Table 4:** Enthalpy variations (ΔH) and peak temperatures of Lutrol F68, Compritol 888 ATO, and lyophilized SLNs.

Sample	ΔH (J/g) Lutrol F68	ΔH (J/g) Compritol 888 ATO	Peak T (°C) Lutrol F68	Peak T (°C) Compritol 888 ATO
**Lutrol F68**	−120.80	----	52.49	----
**SLN G_4_**	−24.58	−106.62	51.06	70.82
**SLN G_3_**	−24.89	−105.73	51.05	70.79
**SLN G_1_**	−50.02	−113.62	51.02	71.26
**SLN G_0_**	−62.64	−114.64	50.06	71.49
**Compritol 888 ATO**	----	−127.67	----	71.64

**Table 5:** Enthalpy variations (ΔH) and peak temperatures of rapidly resuspended SLNs and pre-lyophilization SLN.

Sample	ΔH (J/g)	Peak T (°C)
**SLN G_4_**	−24.58	74.74
**SLN G_3_**	−21.74	74.35
**SLN G_1_**	−23.66	72.86
**SLN G_0_**	−25.82	74.00
**SLN pre-lyo**	−100.86	71.68

**Table 6:** Enthalpy variations (ΔH) and peak temperatures of resuspended SLNs with various methods and pre-lyophilization SLN.

Sample	ΔH (J/g)	Peak T (°C)
**SLN G_1_ vortexed**	−51.58	74.22
**SLN G_1_ manually shaken**	−23.66	72.86
**SLN G_1_ ultrasonicated**	−59.83	71.64
**SLN pre-lyo**	−100.86	71.68

**Table 7:** (looped once to segment 1) DSC scanning program.

Segment	Start Temperature (°C)	End Temperature (°C)	Heating Rate (°C/min)	N_2_ Flow (mL/min)
**1**	25	170	2	70
**2**	170	25	−4	70

**Table 8:** (looped once to segment 1) DSC scanning program.

Segment	Start Temperature (°C)	End Temperature (°C)	Heating Rate (°C/min)	N_2_ Flow (mL/min)
**1**	25	85	2	70
**2**	85	25	−4	70

**Table 9:** DSC scanning program.

Segment	Start Temperature (°C)	End Temperature (°C)	Heating Rate (°C/min)	N_2_ Flow (mL/min)
**1**	25	85	2	70
**2**	85	25	−4	70

## Data Availability

Data is available on the request from the corresponding author.

## Sample Availability

Samples are available from the authors.
